# Salicylic Acid as an Antiseptic, Etc.

**Published:** 1875-11

**Authors:** 


### EDITORIAL. ETC.

---

*Salicylic Acid as an Antiseptic and Disinfectant.*—The Dental and Medical professions have recently heard so much concerning the efficacy of this agent, that considerable interest has been excited, and many experiments made to test it. Those who claim for it all that it required for such an agent, have heretofore met with no opposition to the views they have expressed, but the other side of the question is now appearing, as is shown by the following article from R. Rother, in the October number 1875, of the *London Chemist and Druggist:*

"In spite of the tremendous *furore* that a number of interested writers are making about salicylic acid, the star of this novel and noteworthy substance is even now declining. Most of the glowing reports extolling its wonderful powers were baseless exaggerations, to say nothing of the pure fictions that were manufactured and circulated regarding the disinfecting properties of salicylic acid. The writer had been using benzoic acid as an antiseptic and preservative of certain decomposable solutions with most satisfactory results, as, for instance, in solution of magnesium citrate, both in the form of a concentrated stock solution four times the officinal strength, and the ordinary officinal state; then the 'cream syrup' for soda water, also a concentrated coffee syrup, one to sixteen for soda water, solutions of citric and tartaric acids, and several fermentable officinal syrups. The first application the writer made of salicylic acid was employed more than the water could dissolve. In less than a week the syrup was covered with an extra heavy and exuberant growth of mould and, several days later it passed into active fermentation. The next trial was with red raspberry juice. After the bruised berries had fermented twenty-four hours one drachm of salicylic was added to a quantity of berries representing three gallons of juice; six hours later the magna was pressed and then the fact became apparent that the fermentation was still active—in fact, from the profuse evolution of carbonic acid, it seemed now to be in its most energetic condition. Another drachm of salicylic acid was now added, but six hours later fermentation had not ceased; and, moreover, from the very perceptible acetous odour, the inference was at hand that the acetic fermentation had also begun. One drachm more of salicylic acid was then added, which either stopped the alcoholic fermentation or this ceased simply from the exhaustion of fermentable material. But twelve hours afterwards it was found that the acetic fermentation was raising havoc with the juice, and an additional drachm of salicylic acid was incorporated. It, however, soon became evident that even now the juice was rapidly spoiling, and the writer, in order to save it in its already deteriorated condition, speedily mixed a part of it with four per cent. of alcohol, and converted the remainder into a dense syrup by the addition of sugar.

The writer found that neither salicylic nor benzoic acid will preserve milk, unless added in excess of the natural alkalinity; a comparatively large amount of either will then be required. A mixture of one pint of milk, one ponud of granulated sugar, one fluid drachm of tincture of vanilla, and five to ten grains of benzoic or salicylic acid, makes a very permanent 'cream syrup;' in fact, such a mixture will remain 'sweet' for several days even in the hottest weather, and the writer has not had a single case of 'sour cream' during the whole season. It was, however, found that a small remnant of milk or "cream', remaining in a large bottle became putrid within the space of a week, and, although salicylic acid was added, equal in quantity to this decomposing mass, the offensive effluvium was not diminished in the least, however prolonged the contact might have endured. This circumstance is ample illustration that salicylic acid is by no means a disinfectant.

The conversion of lactin into lactic acid, then into butyric acid, and ultimately into the more odorous products of putrid fermentation, is either checked or entirely obviated in the presence of a perfect antiseptase—that is, any agency which counteracts the effects of septism, and during which action the antiseptase remains indefinitely intact; but an antiseptase, which is also a disinfectant, must finally disappear entirely when placed into an already decomposing body, since the process of disinfection consists partly and importantly in a chemical disintegration of the various elements concerned. Hence, as salicylic acid does not itself decompose in a putrescent mixture, it cannot consequently be a disinfectant, even if it is to a certain extent an antiseptase.

Prof. Salkowsky, of Berlin, has quite recently demonstrated that salicylic acid, in concentrated aqueous solution, will temporarily suspend the appearance of putrefaction, but cannot prevent its occurrence, and that it has no deodorising qualities whatever. If decaying fluids are mixed with salicylic acid the odour still remains entirely unchanged. There is really no apparent reason why salicylic acid should possess deodorising properties, since such action can manifest itself in three different ways, namely: (1) by a destruction of the volatile products of the decaying substance, as, for instance, through the action of potassium permanganate, chlorinated lime and sulphurous acid: (2) by absorption, as with charcoal and in less degree by various other porous materials, to which also belong bodies producing precipitates in albuminous fluids; (3) by concealing the putrid odour, as with carbolic acid. Salicylic acid is not possessed of strong chemical affinities, neither does it produce precipitates, nor has it an inherent odour. The action of salicylic acid does not originate in a splitting up into carbolic and carbonic acids as Kolbe supposed. The inadmissibility of that presumption is apparent from the fact that salicylic acid acts in weaker concentration than carbolic acid. Furthermore, salicylic acid may be recovered from putrid mixtures by means of ether, but carbolic acid is not traceable under the same conditions. Benzoic acid is a much more powerful antiseptic than salicylic acid. Raw meat chopped up fine or in larger pieces, when placed into concentrated solution of benzoic acid, remained perfectly fresh during a period of three months, the liquid remaining perfectly clear and retaining the odour of benzoic acid. The preservation was apparently ab-olute for any period. There is no difference between the effectiveness of natural and artificial benzoic acid.

Kolbe had stated, among numerous other myths about salicylc acid, that it is a much superior antiseptic to benzoic acid. The spirit of pecuniary enterprise is getting a fast hold upon the German chemists. Every new wrinkle is twisted into proportions of wonderful importance, and directly a factory is started, by means of which, and elaborate puffs, the general market is flooded with a worthless, or nearly worthless, substance. About the time the enthused multitude returns to sober second thought, the great scientist is enabled to retire from business to the more secluded pursuits of pure science, with a handsome gain, Kolbe's notorious pamphlet ridiculing the new notation and nomenclature simply because it was of French origin, and because Wurtz, in excessive eulogism, pronounced chemistry a French creation, founded by Lavoisier, is a pretty sample of what can be achieved on the field of pure science.

